# Cognitive screening in treatment-naïve HIV-infected individuals in Hong Kong – a single center study

**DOI:** 10.1186/s12879-019-3784-y

**Published:** 2019-02-13

**Authors:** Fiona C.C. Chan, Phillip Chan, Iris Chan, Andrew Chan, Tommy H. C. Tang, Wilson Lam, W. C. Fong, M. P. Lee, Patrick Li, Germaine H. F. Chan

**Affiliations:** 1 Department of Medicine, Queen Elizabeth Hospital, Hong Kong, SAR People’s Republic of China; 2 SEARCH, Thai Red Cross AIDS Research Centre, 104 Ratchadamri Rd, Khwaeng Pathum Wan, Khet Pathum Wan, Krung Thep Maha Nakhon, Bangkok 10330 Thailand; 3 Department of Clinical Psychology, Queen Elizabeth Hospital, Hong Kong, SAR People’s Republic of China; 4 Hong Kong Sanatorium and Hospital, Hong Kong, SAR People’s Republic of China

**Keywords:** HIV, cART naïve, Cognitive screening, MoCA, IHDS, Hong Kong

## Abstract

**Background:**

HIV-associated neurocognitive disorder (HAND) remains prevalent in the era of combination antiretroviral therapy (cART). The prevalence of HAND in Hong Kong is not known.

**Methods:**

Between 2013 and 2015, 98 treatment-naïve HIV-1-infected individuals were referred to and screened by the AIDS Clinical Service, Queen Elizabeth Hospital with (1) the International HIV Dementia Scale (IHDS), a screening tool that targets moderate to severe HAND, (2) the Montreal Cognitive Assessment (MoCA), a frequently used cognitive screening test and (3) the Patient Health Questionnare-9 (PHQ-9), a 9-item questionnaire that evaluates depression symptoms. Within the study period, 57 of them completed the second set of IHDS and MoCA at 6 months after baseline assessment.

**Results:**

Most participants were male (94%), with a median age of 31 years. At baseline, 38 (39%) and 25 (26%) of them scored below the IHDS (≤10) and MoCA (25/26) cut-offs respectively. Poor IHDS performers also scored lower on MoCA (*p* = 0.039) but the correlation between IHDS and MoCA performance was weak (r = 0.29, *p* = 0.004). Up to a third of poor IHDS performers (13/38) showed moderate depression (PHQ-9 > 9). In the multivariable analysis, a lower education level (*p* = 0.088), a history of prior psychiatric illness (*p* = 0.091) and the presence of moderate depression (*p* = 0.079) tended to be significantly associated with poor IHDS performance.

At follow-up, 54 out of 57 were on cART, of which 46 (85%) had achieved viral suppression. Their blood CD4+ T-lymphocytes and IHDS scores were higher at follow-up compared to baseline values (both *p* < 0.001) but their MoCA performance was similar at both assessments. Of note, 17 participants in this subgroup scored below the IHDS cut-off at both assessments.

**Conclusions:**

Poor IHDS performance, and likely cognitive impairment, was frequently observed in treatment-naïve HIV-infected individuals in our locality. A considerable proportion continued to score below the IHDS cut-off at 6 months after cART. Depression was frequently observed in this vulnerable population and was associated with poor IHDS performance.

**Electronic supplementary material:**

The online version of this article (10.1186/s12879-019-3784-y) contains supplementary material, which is available to authorized users.

## Background

The introduction of combination antiretroviral therapy (cART) has changed HIV-1 infection from a life-threatening disease to a manageable chronic condition, and life expectancy of people living with HIV (PLWH) is approaching that of non-infected individuals [1]. However, the impact of cART is less significant in the central nervous system (CNS). While CNS opportunistic infections are rare, cognitive impairment remains common in PLWH on cART [2–4]. It is estimated that the frequency of HIV associated dementia (HAD), the most severe form of HIV associated cognitive disorder (HAND), has dropped from 20 to 2% among PLWH in the cART era. However, the rate of milder forms of HAND still ranges from 15 to 55% in different cohorts [2–4].

Apart from the impact on work performance and quality of life, cognitive impairment in PLWH could affect drug adherence and hence virological control [5]. In Hong Kong, the population of PLWH exceeded 8000 in 2016 with around 700 new cases reported each year and the prevalence of cognitive impairment in this vulnerable population is largely unknown. In a multi-center cross-sectional study of 10 Asia-Pacific regions in 2008, cognitive impairment was reported in 12% of 647 clinic-followed PLWH, but this percentage was 23% among the 61 Hong Kong participants [6]. Estimating the local prevalence of cognitive impairment is essential for health funding, resource allocation and patient support.

This study aimed to estimate the frequency of possible cognitive impairment in cART-naïve HIV-infected individuals referred to HIV-clinic service in Hong Kong. We compared cognitive screening test performance before and after cART and identified potential risk factors that were associated with poor test performance.

## Methods

### Study design and participants recruitment

Participants included all consecutive treatment-naïve referrals to the AIDS Clinical Service, Queen Elizabeth Hospital, Hong Kong, between October 1, 2013 and October 31, 2015. Only ethnic Chinese were included to ensure standardization of population and the cognitive screening procedures. Individuals with a known history of major medical or neurological disorders were excluded. At enrollment, the participants underwent clinical assessment, cognitive and depression screening, and laboratory tests. They were reassessed approximately six months later. All participants received written information sheets for the study and provided verbal informed consent. This study was approved by the Kowloon Central/Kowloon East Research Ethics Committee of Hospital Authority, Hong Kong (Reference number: KC/KE-14-0065/FR-1).

### Clinical and laboratory data acquisition

At baseline, demographic data, social history and past medical history, including route of HIV-1 transmission, were collected. Laboratory tests included blood CD4+ T-lymphocytes level, HIV-1 subtyping, HIV-1 ribonucleic acid (RNA) quantification, and serology of syphilis and viral hepatitis (B and C). Blood CD4+ T-lymphocytes and HIV-1 RNA levels were tested again in parallel with cognitive screening at follow-up. Individual cART regimens were recorded based on the CNS penetration-effectiveness (CPE) index [7]: a high CNS penetrating regime was defined as CPE > 7.

### Cognitive function and depression assessment

A formal diagnosis of HAND relies on detailed neuropsychiatric and functional status assessment conducted by a clinical psychologist (CP) [3] but such assessments require a significant amount of time and resources. The International HIV dementia scale (IHDS) is a validated brief 3-min screening test that is ideal for this pilot study which aims to estimate the frequency of cognitive impairment in newly referred patients. IHDS examines motor speed, psychomotor speed and memory-recall function [8] and a score of ≤10 (out of 12) shows a reasonable sensitivity (64–74%) and specificity (55–66%) in identifying moderate to severe HAND [9]. The Hong Kong version of the Montreal Cognitive Assessment (MoCA) was added to supplement the breadth of cognitive screening (visuospatial/executive, naming, memory, attention, language, abstraction, delayed recall, and orientation). In our locality, a cut-off of 21/22 (out of 30) has been validated for evaluating mild cognitive impairment in elderly and stroke patients [10, 11], while a cut-off of 25/26 is used in the original English version and previous HAND-related studies in Asian societies including Singapore [12] and Korea [13, 14]. Both cut-off values were included in the results for reference.

At both baseline and follow-up assessments, participants were screened with IHDS and MoCA by CP or trained research assistants. At baseline, participants also completed the Patient Health Questionnare-9 (PHQ-9), a 9-item questionnaire which scores each of the 9 DSM-IV criteria from “0” (not at all) to “3” (nearly every day) over the previous 2 weeks [15]. The Chinese version of PHQ-9 has been validated in Hong Kong [16], with a cut-off score > 9 for moderate depression [15].

### Statistical analysis

Continuous variables were presented as median and interquartile ranges (IQR). Pearson’s Chi-squared test and Fisher’s exact test were used for categorical variables. Non-parametric Mann-Whitney U test was used for continuous variables. Multivariable logistic regression models were fitted to identify baseline factors associated with poor IHDS performance (cut-off: < 10). McNemar and Wilcoxon signed-rank test were used to compare variables before and after cART accordingly. A *p*-value < 0.05 was considered significant. Statistical analyses were performed using SPSS Version 20.0 (International Business Machines Corporation, New York, USA).

## Results

During the study period, 102 patients were referred, of whom 4 individuals were excluded from participation: two were cART-experienced, one had a CNS opportunistic infection, and one had intellectual disability. None were lost to follow-up but one participant succumbed before follow-up due to a non-HIV-related illness. Fifty-seven completed the follow-up assessment within the study period.

Of the 98 enrolled individuals (Table 1), more than a third (*n* = 37, 38%) were referred from our in-patient service for continuation of care. Most were male (*n* = 92, 94%) with a median age of 31 years (IQR 26–43). Sexual contact among men who have sex with men (MSM) was the predominant route of HIV acquisition (77%). HIV-1 subtypes CRF01_AE (42%) and B (37%) were the predominant strains. Eighteen (18%) had a previous AIDS-defining illness. The median pre-cART blood HIV-1 RNA level was 5.07 (IQR 4.68–5.47) log_10_ copies/mL and the median nadir CD4+ T-lymphocyte level was 270 (IQR 106–376) cells/μL.

**Table 1:** Demographic and Clinical Characteristics of Participants at Baseline (*N* = 98)

Male, *n* (%)	92 (94)
Female, *n* (%)	6 (6)
Age, year	31 (26–43)
Tertiary education, *n* (%)	49 (50)
Current Smoker, *n* (%)	31 (32)
Current or ex-drinker, *n* (%)	22 (22)
History of substance Use, *n* (%)	41 (42)
Prior psychiatric illness, *n* (%)	14 (14)
Route of transmission, *n* (%)	
MSM	75 (77)
Other	23 (23)
HIV-1 subtype, *n* (%)	
CRF01_AE	41 (42)
B	36 (37)
Other	21 (21)
AIDS^a^, *n* (%)	18 (18)
Blood CD4+ T-cells nadir^b^ (cells/μL)	270 (106–376)
Blood HIV-1 RNA, log10 copies/ml	5.07 (4.68–5.47)
Hepatitis B virus co-infection, *n* (%)	6 (6)
Hepatitis C virus co-infection, *n* (%)	7 (7)
Syphilis co-infection, *n* (%)	40 (41)
Recent in-patient care, *n* (%)	37 (38)
IHDS ≤10, *n* (%)	38 (39)
MoCA ≤25, *n* (%)	25 (26)
MoCA ≤21, *n* (%)	8 (8)
MoCA score	27 (25–28)
Moderate Depression (PHQ-9 > 9)^b^, *n* (%)	23 (24)

Median (IQR) is presented unless specified otherwise

^a^Defined by the presence of AIDS-defining illness regardless of CD4+ T-lymphocyte levels

^b^*n* = 96

Abbreviations: *IHDS* International HIV Dementia Scale, *MoCA* Montreal Cognitive Assessment, *PHQ-9* Patient Health Questionnare-9, *MSM* men who have sex with men

### Risk factors of poor IHDS performance at baseline

At baseline, 38 participants (39%) scored ≤10 on IHDS. Their demographic and clinical characteristics were compared to those who scored above this cut-off **(**Table 2**)**. In the univariable analyses, poor IHDS performers had higher rates of prior psychiatric illness (24% vs. 8%, *p* = 0.034) and moderate depression (PHQ-9 > 9) (34% vs. 17%, *p* = 0.048). They tended to have lower education level (40% vs. 57% tertiary education, *p* = 0.097) and blood CD4+ T-lymphocyte counts (241 (IQR 69–320) vs. 298 (IQR 160–400), *p* = 0.078). Age, sex, smoking, alcohol and substance use, blood HIV-1 RNA level, syphilis, and hepatitis C co-infection did not associate with poor IHDS performance (all *p* > 0.1). In the multivariable analysis, prior psychiatric illness (adjusted odds ratio (aOR) 2.99; 95% CI 0.84–10.66, *p* = 0.091), moderate depression (aOR 2.52; 95% CI 0.90–7.06, *p* = 0.079) and lower education level (tertiary education: aOR 0.47; 95%CI 0.20–1.17, *p* = 0.088) tended to be significantly associated with poor IHDS performance, while CD4+ T-lymphocyte level was no longer significant.

**Table 2:** Factor Associated with Cognitive Impairment Status According to IHDS Score

			Univariable Analysis		Multivariable Analysis	
	Non-impaired (IHDS > 10) (*n* = 60)	Impaired (IHDS ≤10) (*n* = 38)	Odds Ratio (95% CI)	*p*-value	Adjusted Odds Ratio (95% CI)	*p*-value
^a^Female sex, n (%)	3 (5)	3 (8)	1.63 (0.31–8.52)	0.674		
Age, year	31 (25–40)	32 (26–45)		0.358		
^b^Tertiary education, *n* (%)	34 (57)	15 (40)	0.50 (0.22–1.14)	0.097	0.47 (0.20–1.17)	0.088
^b^Current smoker, *n* (%)	19 (32)	12 (32)	1.00 (0.44–2.25)	1.000		
^b^Current or ex-drinker, *n* (%)	14 (23)	8 (21)	0.88 (0.33–2.34)	0.792		
^b^History of substance use, *n* (%)	23 (38)	18 (47)	1.45 (0.64–3.30)	0.377		
^b^Prior psychiatric illness, *n* (%)	5 (8)	9 (24)	3.41 (1.05–11.13)	0.034	2.99 (0.84–10.66)	0.091
Blood HIV-1 RNA, log_10_ copies/ml	5.01 (4.61–5.39)	5.11 (4.81–5.75)		0.139		
CD4+ T-cells nadir (cells/μL)	298 (160–400)	241 (69–320)		0.078		NS
^b^CD4+ T-cells nadir < 200 cells/μL, *n* (%)	18 (30)	13 (34)	1.21 (0.51–2.89)	0.662		
^a^Hepatitis C virus co-infection, *n* (%)	5 (8)	2 (5)	0.61 (0.11–3.32)	0.703		
^b,c^ Syphilis co-infection, *n* (%)	22 (37)	18 (47)	1.56 (0.68–3.55)	0.294		
^d^PHQ-9 score	5 (2–8)	8 (4–12)		0.127		
^b,d^Moderate depression (PHQ-9 > 9), *n* (%)	10 (17)	13 (34)	2.67 (0.99–7.17)	0.048	2.52 (0.90–7.06)	0.079

Median (IQR) is presented unless specified otherwise

^a^Fisher’s Exact test

^b^Pearson Chi-square test

^c^History of infection (enzyme immunoassay (EIA)-Treponemal pallidum assay (TPA) positivity)

^d^*n* = 96

Abbreviations: *IHDS* International HIV Dementia Scale, *MoCA* Montreal Cognitive Assessment, *PHQ-9* Patient Health Questionnare-9, *NS* Not significant

### Correlation between IHDS and MoCA performance at baseline

**Fig. 1:**
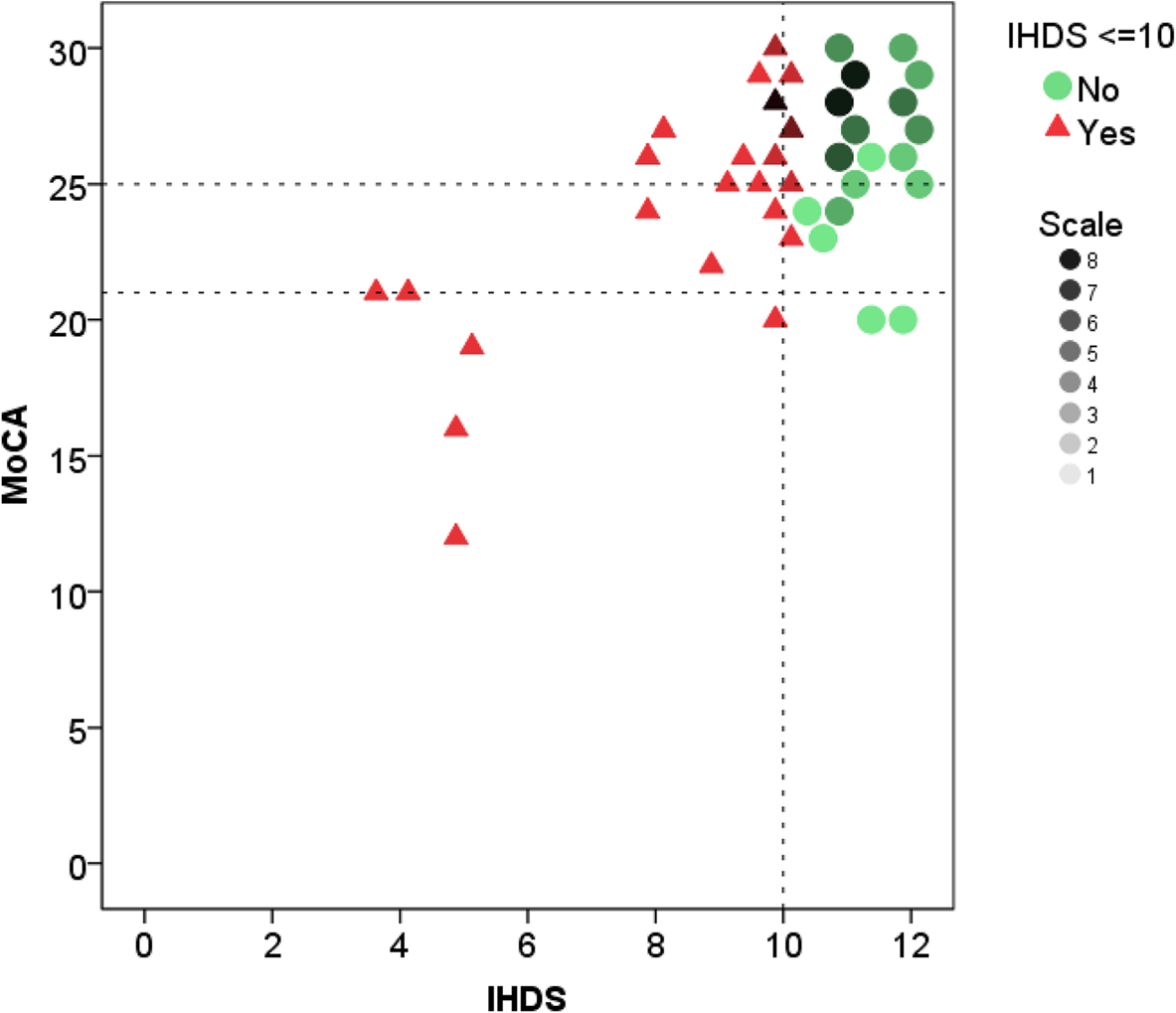
Participants’ Performance in IHDS and MoCA at Baseline. Dotted lines: cut-offs of MoCA (≤21 and ≤ 25) and IHDS (≤10)

Poor IHDS performers also performed worse on the MoCA compared to those who scored above the IHDS cut-off (27 (IQR 24–28) vs. 28 (IQR 26–29), *p* = 0.039). Participants’ IHDS scores were only weakly correlated with their MoCA performance (Bivariate correlation (Spearman’s): r = 0.29, *p* = 0.004). In particular, nearly one third (*n* = 24) of the 73 participants who scored above the MoCA cut-off (> 25) scored below the IHDS cut-off. The participants’ IHDS and MoCA scores are illustrated in Fig. 1.

### Clinical and cognitive screening outcomes at 6 months follow-up

Within the study period, 57 participants completed their second assessment, approximately 6 months after the baseline assessment. Compared to those who did not yet complete the second assessment, this subgroup of participants had a lower baseline IHDS score (10 (IQR 10–11) vs. 11 (IQR 11–12), *p* < 0.001). They also had a high proportion of poor IHDS performers (58% vs 12%, *p* < 0.001). However, both subgroups were statistically similar in terms of demographic and clinical parameters except that the subgroup with follow-up had a higher blood HIV-1 RNA at baseline (5.11 vs 4.91 log_10_ copies/ml, *p* = 0.010) (Additional file 1: Table S1).

Fifty-four of the 57 participants who completed both assessments were on cART (Table 3) and 46 (85%) had achieved viral suppression at follow-up (blood HIV-1 RNA < 20 copies/ml). Their blood CD4+ T-lymphocyte level and IHDS performance both improved compared to baseline (468 (IQR 261–673) vs. 248 (IQR46–355) cells/mm3, p < 0.001 and 11 (IQR 10–12) vs. 10 (IQR 10–11), p < 0.001 respectively). None of them converted from above to below the IHDS cut-off at follow-up and their MoCA test performances were statistically similar between two assessments (*p* > 0.1). Seventeen out of thirty-one participants who scored below the IHDS cut-off at baseline again scored below the cut-off at follow-up.

**Table 3:** Clinical and Cognitive Outcomes of the Subset of Participants with Follow-up (*n* = 54)^a^

	Baseline	Follow-up	*p*-value
CD4+ T-lymphocyte (cells/μL)	248 (46–355)	468 (261–673)	< 0.001
On cART with CPE > 7, *n* (%)	–	7 (13)	–
HIV-1 RNA suppression^b^, *n* (%)	–	46 (85)	–
IHDS score	10 (10–11)	11 (10–12)	< 0.001
IHDS ≤10, *n* (%)	31 (57)	17 (32)	< 0.001
MoCA score	27 (26–29)	27 (26–28)	0.818
MoCA ≤25, *n* (%)	13 (24)	12 (22)	1.000
MoCA ≤21, *n* (%)	4 (7)	4 (7)	1.000

Median (IQR) is presented unless specified otherwise

^a^Only included participants who were on cART

^b^Blood HIV-1 RNA level < 20 copies/mL

Abbreviations: *IHDS* International HIV Dementia Scale, *MoCA* Montreal Cognitive Assessment, *CPE* CNS penetration-effectiveness

Linear regression was employed to determine factors that were associated with the change in IHDS scores (i.e. follow-up IHDS score minus baseline IHDS score) (Additional file 2: Table S2). In the univariable analysis, older age was associated with IHDS improvement (Mean difference 0.03, 95% CI (0.01 to 0.06), *p* = 0.013). Tertiary education and history of prior psychiatric illness, which tended to be significantly associated with IHDS performance at baseline, were not associated with IHDS change (*p* > 0.1). HIV-related factors, including plasma CD4+ T-lymphocytes nadir, viral suppression status, and CPE index of cART were not associated with change of IHDS scores (*p* > 0.1).

## Discussion

This study estimated the frequency of possible cognitive impairment based on an IHDS cut-off (≤10) that targets more severe forms of HAND. Recent studies suggest a higher cut-off of ≤11 to improve the IHDS sensitivity towards milder forms of HAND [17, 18]. At baseline, up to 40% of this group of relatively young, male predominant, cART-naïve individuals scored below the IHDS cut-off. In the MoCA test that examined a different set of cognitive domains, 26 and 8% scored below the original English version (25/26) and locally validated (21/22) cut-offs, respectively. Of note, six participants scored below both IHDS and MoCA 21/22 cut-offs, suggesting multi-domain cognitive impairment. Poor IHDS performers also scored lower on the MoCA but the correlation was weak. Up to a third of participants with normal MoCA scores (> 25) scored below the IHDS cut-off, suggesting a discrete impairment in motor, psychomotor and memory-recall functions without a major involvement in cognitive domains examined by MoCA. Thus, a stand-alone MoCA test would be insufficient for cognitive screening in this group of individuals.

The subgroup of participants who completed the second cognitive screening within the study period had poorer IHDS performance and higher blood HIV-1 RNA levels than the remaining participants while their MoCA performance and clinical parameters were similar. The higher rate of poorer IHDS performers in this subgroup seems random, rather than driven by HIV-1 RNA level, as HIV-1 RNA was not associated with IHDS performance in the multivariable analysis at baseline.

At follow-up, this subset of participants showed an improvement in IHDS performance after cART, but the increment in score was modest. Moreover, a considerable proportion of these participants again scored below the IHDS cut-off at follow-up. Improvement in MoCA test performance was also absent. The overall lack of improvement in both tests could be due to irreversible neurocognitive impairment, driven by HIV-1 infection and/or other etiologies. Other possibilities include the insensitivity of IHDS and MoCA to detect cognitive improvement or a delayed onset of cognitive improvement up to 9 months after cART [19, 20].

At baseline, education, prior psychiatric illness and co-existing moderate depression (PHQ-9 > 9), but not HIV-specific parameters (HIV-1 RNA level and CD4+ T-lymphocytes nadir) [2, 4, 21] or co-infections (syphilis and HCV) [22, 23], tended to be independently associated with poor IHDS performance. The association between IHDS performance and education, particularly years of education, was previously highlighted in another study [17]. Better education is generally considered as a protective factor against cognitive impairment, likely due to a better cognitive reserve that contributes to resilience against neuropathological insults [24].

The association between mood disorders and cognitive impairment is frequently reported. In particular, major psychiatric illnesses including depression are linked to long term structural brain changes [25] and cognitive decline [26]. Depression is frequently observed in HIV-infected populations [27] and is also a risk factor for HAND [21]. Differentiating the effect of depression from HAND in neurocognitive assessment is challenging and requires detailed neuropsychiatric assessment [28]. However, none of the factors that were associated with baseline IHDS performance nor HIV-related parameters or CPE index of cART were associated with the change of IHDS performance after cART. This general lack of association could be related to the aforementioned irreversible cognitive impairment, insensitivity of the IHDS, or brief interval between assessments for observing the benefit of cART on neurocognitive functioning.

We observed poor IHDS performance in 39% of treatment-naïve HIV-infected study participants, a frequency based on a cut-off that targets for more severe forms of HAND. Despite the usefulness of MoCA in degenerative neurocognitive diseases, MoCA alone may not be an ideal screening tool in HIV-infected populations because of the limited correlation with IHDS outcomes. A considerable proportion of our participants had concomitant moderate depression symptoms (PHQ-9 > 9), which tended to be independently associated with poor IHDS performance. Despite cART resulting in virological control in the majority of the group with follow-up, poor IHDS performance persisted in a sizable proportion of them. The findings highlight the need for comprehensive allied health support in contemporary HIV care, including cognitive and mood assessment, and cognitive rehabilitation may be needed. Hong Kong has a relatively young HIV-infected population with a median age under 40. As this population of PLWH ages due to the improved survival and an as yet stable number of newly reported cases, the service demand for cognitive and mood disorders is expected to increase.

Our study has its limitations. First, although the co-dominant HIV-1 B and CRF_01AE subtypes in our participants is compatible with the local HIV-1 strains pattern [29], they had a relatively high rate of recent hospitalization and might not fully match the new local HIV-1 cases in terms of disease severity. The frequency of AIDS in this study was 18%, compared to 14% (218/1558) among total newly reported cases for the corresponding period in Hong Kong (HIV Surveillance Report 2014 and 2015, Centre for Health Protection, Department of Health). Second, repeating cognitive tests at 6 months after cART could be too early to observe cognitive improvement [19, 20]. Third, the higher rate of poorer IHDS performers at baseline among the subgroup with follow-up assessment may lead to overestimation of the frequency of persistent cognitive impairment after cART. Lastly, cognitive outcomes in this study were estimated by IHDS and MoCA, which are designed for screening purposes. The lack of HIV-negative controls limits the tests’ validity in estimating the frequency of cognitive impairment of the study participants.

## Conclusion

Our study suggests that moderate to severe cognitive impairment exists in a considerable proportion of treatment-naïve PLWH in Hong Kong. The population of PLWH in Hong Kong is relatively young and is going to expand further. Our findings support the need for implementing cognitive and mood disorder assessments in routine HIV clinical care. This approach may reduce longer term neurocognitive impairment and alleviate its socioeconomic consequences.

## Additional files

Additional file 1: **Table S1.** Demographic and Clinical Characteristics of Participants with and without Second Cognitive Screening. (DOCX 15 kb)

Additional file 2: **Table S2.** Factor Correlation with IHDS Changes. (DOCX 14 kb)
