# Supplementary material for: Cognitive screening in treatment-naïve HIV-infected individuals in Hong Kong – a single center study
Source: BMC Infect Dis. 2019 Feb 13;19:156. doi: 10.1186/s12879-019-3784-y (PMC6375138; doi:10.1186/s12879-019-3784-y)
Supplement: Supplementary file 1 — Table S1. Demographic and Clinical Characteristics of Participants with and without Second Cognitive Screening. (DOCX 15 kb) [file 12879_2019_3784_MOESM1_ESM.docx]

| **Additional file 1: Table S1. Demographic and Clinical Characteristics of Participants with and without Second Cognitive Screening** | | | |
| --- | --- | --- | --- |
|  | **Participants with Second Cognitive Screening**  **(n=57)** | **Participants without Second Cognitive Screening**  **(n=41)** | **p-value** |
| ***Female Sex, n (%)** | 2 (4) | 4 (10) | 0.233 |
| **Age, year** | 31 (26-45) | 29 (24-40) | 0.219 |
| **^#^Tertiary education, n (%)** | 26 (46) | 23 (56) | 0.306 |
| **^#^Current smoker, n (%)** | 19 (33) | 12 (29) | 0.669 |
| **^#^Current or ex-drinker, n (%)** | 10 (18) | 12 (29) | 0.260 |
| **^#^History of substance Use, n (%)** | 26 (46) | 15 (37) | 0.371 |
| **^#^Prior psychiatric illness, n (%)** | 9 (16) | 5 (12) | 0.834 |
| **Blood HIV-1 RNA, log_10_ copies/ml** | 5.11 (4.90-5.63) | 4.91 (4.52-5.35) | 0.010 |
| **CD4+ T-cells nadir (cells/µL)** | 267 (60-361) | 272 (178-437) | 0.130 |
| **CD4+ T-cells nadir < 200 cells/µL** | 20 (35) | 11 (27) | 0.386 |
| ***Hepatitis C virus co-infection, n (%)** | 3 (5) | 4 (10) | 0.447 |
| **^#&^Syphilis co-infection, n (%)** | 24 (42) | 16 (39) | 0.760 |
| **IHDS score** | 10 (10-11) | 11 (11-12) | <0.001 |
| **IHDS ≤ 10, n (%)** | 33 (58) | 5 (12) | <0.001 |
| **MoCA ≤ 25, n (%)** | 13 (23) | 12 (29) | 0.469 |
| **MoCA ≤ 21, n (%)** | 4 (7) | 4 (10) | 0.716 |
| **MoCA score** | 27 (26-29) | 27 (25-28) | 0.290 |
| **^#^^Moderate depression (PHQ-9>9), n (%)** | 15 (26) | 8 (20) | 0.588 |
| Median (IQR) is presented unless specified otherwise.  ^^^ n = 96; ^#^ Pearson Chi-square test; * Fisher’s Exact test.  Abbreviations: IHDS = International HIV Dementia Scale, MoCA = Montreal Cognitive Assessment; PHQ-9 = Patient Health Questionnare-9 | | | |
