# Supplementary material for: Cognitive screening in treatment-naïve HIV-infected individuals in Hong Kong – a single center study
Source: BMC Infect Dis. 2019 Feb 13;19:156. doi: 10.1186/s12879-019-3784-y (PMC6375138; doi:10.1186/s12879-019-3784-y)
Supplement: Supplementary file 2 — Table S2. Factor Correlation with IHDS Changes. (DOCX 14 kb) [file 12879_2019_3784_MOESM2_ESM.docx]

| **Additional file 2: Table S2: Factor Correlation with IHDS Changes*** | | |
| --- | --- | --- |
|  | **IHDS mean difference (95% CI)** | **p-value** |
| **Female sex** | 0.78 (-087 to 2.45) | 0.345 |
| **Age, year** | 0.03 (0.01 to 0.06) | 0.014 |
| **Tertiary education** | -0.17 (-0.81 to 0.46) | 0.592 |
| **Current smoker** | -0.04 (-0.40 to 0.31) | 0.811 |
| **Current or ex-drinker** | 0.24 (-0.60 to 1.09) | 0.565 |
| **History of substance use** | -0.17 (-0.81 to 0.46) | 0.592 |
| **Prior psychiatric illness** | -0.62 (-1.45 to 0.21) | 0.139 |
| **HIV-1 RNA suppression** | -0.08 (-0.98 to 0.81) | 0.850 |
| **CPE index ≥ 8** | 0.13 (-0.81 to 1.08) | 0.777 |
| **CD4+ T-cells nadir, per 100 cells/µL** | -0.1 (-0.3 to 0.05) | 0.135 |
| **CD4+ T-cells nadir < 200 cells/µL** | 0.45 (-0.19 to 1.10) | 0.165 |
| **Hepatitis C co-infection** | -0.26 (-1.63 to 1.13) | 0.712 |
| **Syphilis co-infection** | -0.25 (-0.89 to 0.39) | 0.432 |
| *IHDS change = Follow-up IHDS score minus Baseline IHDS score  Statistical method: Linear regression with IHDS change as dependent variable  Abbreviation: CPE = CNS Penetration-Effectiveness | | |
